# Expression of the NEK family in normal and cancer tissue: an immunohistochemical study

**DOI:** 10.1186/s12885-019-6408-4

**Published:** 2020-01-06

**Authors:** Talita Diniz Melo-Hanchuk, Mariana Bonjiorno Martins, Lucas Leite Cunha, Fernando Augusto Soares, Laura Sterian Ward, José Vassallo, Jörg Kobarg

**Affiliations:** 1 Departamento de Bioquímica e de Biologia Tecidual, Instituto de Biologia, Universidade Estadual de Campinas, Campinas, São Paulo Brazil; 2 Laboratório de Genética Molecular do Câncer, Faculdade de Ciências Médicas Universidade Estadual de Campinas, Campinas, São Paulo Brazil; 3 Departamento de Patologia, A.C.Camargo Cancer Center, São Paulo, São Paulo, Brazil; 4 Departamento de Anatomia Patológica, Faculdade de Ciências Médicas, Universidade Estadual de Campinas, Campinas, São Paulo Brazil; 5 Faculdade de Ciências Farmacêuticas-UNICAMP, Universidade Estadual de Campinas, Campinas, Inst. de Biologia, Dep. Bioquímica e Biologia Tecidual, Rua Monteiro Lobato 255, CEP 13083-862, Campinas-SP, Brazil

**Keywords:** Tissue mirco array, Thyroid cancer, NEK kinase family, Cancer, Diagnosis, Prognosis, NEK1, NEK3, NEK5

## Abstract

**Background:**

The NEK serine/threonine protein kinases are involved in cell cycle checkpoints, DNA damage repair, and apoptosis. Alterations in these pathways are frequently associated with cell malignant cellular transformations. Thyroid cancer is the most common malignant tumour in the endocrine system. Despite good treatment methods, the number of cases has increased significantly in recent years. Here, we studied the expression of NEK1, NEK2, NEK3, and NEK5 in different types of normal and malignant tissues, using tissue microarray analysis, and identified NEKs as potential markers in thyroid malignancy.

**Methods:**

The studied cases comprised multiple cancer tissue microarrays, including breast, colon, esophagus, kidney, lung, pancreas, prostate, stomach, thyroid and uterine cervix, as well as 281 patients who underwent thyroid resection for thyroid cancer or thyroid nodules. The expression of NEK1, NEK2, NEK3, and NEK5 was analyzed by immunohistochemistry. The expression pattern was evaluated in terms of intensity by two methods, semiquantitative and quantitative, and was compared between normal and cancer tissue.

**Results:**

We analysed the expression of each member of the NEK family in a tissue-dependent manner. Compared to normal tissue, most of the evaluated proteins showed lower expression in lung tumour. However, in the thyroid, the expression was higher in malignant tissue, especially for NEK 1, NEK3 and NEK5. Concerning characteristics of the thyroid tumour, such as aggressiveness, NEK1 expression was higher in tumours with multifocality and in patients with lymph node metastasis. NEK3 expression was stronger in patients with stage II, that involved metastasis. NEK5, on the other hand, showed high expression in patients with invasion and metastasis and in patients with tumour size > 4 cm. Furthermore, this work, demonstrated for the first time a high specificity and sensitivity of over-expression of NEK1 in classical and follicular variants of papillary thyroid cancer and NEK3 in tall-cell papillary thyroid cancer.

**Conclusion:**

Taken together, the NEK protein kinases emerge as important proteins in thyroid cancer development and may help to identify malignancy and aggressiveness features during diagnosis.

**Trial registration:**

This study was retrospectively registered.  www.accamargo.org.br/cientistas-pesquisadores/comite-de-etica-em-pequisa-cep.

## Background

Protein kinases (PKs) mediate most of signal transduction events in eukaryotic cells, either by altering substrate activity or location, interacting with other proteins or by controlling metabolism, transcription, cell cycle progression, cytoskeletal rearrangement, cellular movement, apoptosis and differentiation [1]. PKs regulate the cell cycle, especially at the checkpoint process, are considered interesting therapeutic targets in cancer [2]. Failures at these control points may lead to the development of tumour cells that show altered division rates and may present accumulation of DNA errors (for review [3]). In a large-scale screening of in situ hybridization on tissue microarrays, around 20% of the serine/threonine kinases analysed exhibited altered levels of transcripts in tumours [4].

Of those PKs functionally involved in regulating the cell cycle, and its checkpoints, the members of the 11 NIMA-related kinases (NEK-family), are possibly among the least studied and functionally enigmatic kinases. In this NEK family, 11 genes are encoding different serine/threonine kinases, which catalytic domains have 40–45% amino acid sequence identity with NIMA’s catalytic domain [5]. They have been localised to the cilia, centrosomes, nucleus, cytoplasm, and mitochondria [6].

NEK1, was reported to display, an in vitro,dual serine-threonine and tyrosine kinase activity [7]. Genetic loss-of-function mutations in the NEK1 gene, cause expression of a truncated, functionally inactive, protein kinase. When this mutation is germline derived from birth on, it causes the development, later in life, of the so-called “Polycystic Kidney Disease”. NEK1 has been further associated with bladder [8], kidney [9] and breast [10]cancers.

Similarly, NEK3 [11] and NEK2 [12] were found to be over-expressed, in human breast cancer. Human NEK3 appears to play an important role in prolactin receptor signaling, specifically in a pathway that contributes to the progression of breast cancer and increases the motility of breast cancer cells in vitro [11]. The expression of the full-length NEK3 protein is higher in prostate cancer samples compared to normal controls, but, to the contrary, normal samples have a higher expression of the shorter protein isoform [13].

RNA-seq. Transcriptome analyses of prostate cancer samples, benign, prostatic hyperplasia, and normal tissue, revealed, NEK5 and NEK2 are over-expressed in malignant tissue [14]. NEK2, is also related to progression and poor prognosis in prostate cancer [15, 16].

With a rapidly increasing incidence around the world, thyroid cancer (TC) represent the most common endocrine malignancy. From the 53.990 new diagnoses reported in the United States in 2018 and approximately 75% were associated with women [17, 18]. A rise in incidence numbers over the years is partly explained by the improvement in diagnosis. The majority of the thyroid tumours are classified as well-differentiated papillary (PC) and follicular carcinoma (FC) subtypes (85%), but anaplastic thyroid (AC) and medullary thyroid carcinomas (MC) are also classified.

The development, progression, invasion, and metastasis of TC is related to multiple signaling pathways, such as: JAK/STAT, Wnt-β-catenin, NF-κB, TSHR and PI3K/AKT [19]. TC has a relatively good cure rate, especially when detected at the early stages. However, approximately 20% of the patients tend to not respond to therapy. Therefore, improved diagnostics, alternative treatments and novel target proteins for these non-responsive patients are highly desired.

The objective of the present study was to understand the involvement of four members of the NEK family (NEK1, NEK2, NEK3 and NEK5) in different types of cancers by analyzing differential expression profiles through tissues microarray (TMA) assays. The present study also aimed to investigate a possible role for NEK1, NEK3, and NEK5 in thyroid malignancy, and analyze their potential as diagnostic and prognostic markers.

## Methods

### Cancer specimens and thyroid cancer patients

The first studied cases were multiple cancer tissue microarrays (TMAs) with 10 common types of cancer (breast, colon, esophagus, kidney, lung, pancreas, prostate, stomach, thyroid and uterine cervix) in quadruplicates of each with matched normal tissue and duplicated cores per case (except single cores for the prostate cases). These tissue microarrays were purchased from US Biomax Inc. (MC802 from US Biomax Inc., Rockville, MD, USA).

Also, we studied 281 patients who underwent thyroid resection for thyroid nodules of different histologic types; thyroid carcinoma was diagnosed in 193 patients: 168 were papillary thyroid carcinomas (PTC) and 25 follicular thyroid carcinomas (FTC). The most frequent histotype among papillary carcinomas was the classic variant (CPTC) (107 cases), followed by the follicular variant (FVPTC) (48 cases) and the tall-cell (TCPTC) variant was identified in 13 cases. We also obtained benign tissue from patients, including 31 follicular adenomas (FA) and 42 goiters (G), and 15 normal thyroid tissues obtained from the contralateral lobe of FA (Normal Tissue). The American Joint Committee on Cancer (AJCC) TNM system was used to detect aggressiveness at diagnosis for differentiated thyroid carcinomas. Patients were managed according to Latin American Thyroid Society (LATS) and Ameican Thyroid Association (ATA) guidelines and followed for a period of 12 to 175 months (median = 35; mean = 41.1; SD [standard deviation] = 26 months). All the clinical, surgical, and pathological reports, also as follow-up data, were recorded.

For diagnostic confirmation, two experienced pathologists (JV and FAS) reviewed carefully and independently all tumours. Cases presenting conflicting results or areas of poor differentiation were excluded. Paraffin blocks of formalin-fixed tissues were collected and, in each case, the most representative area of the tumour, normal surrounding tissue, tumour areas of invasiveness, and metastatic tissue were selected and micro-dissected, whenever available. The Research Ethics Committees of the AC Camargo Cancer Center, São Paulo, Brazil (1259/09-C), approved this study protocol.

### Immunohistochemical detection

Commercial TMA slides of multiple cancer types were purchased from US Biomax Inc. (Rockville, Maryland, USA). Thyroid TMA was constructed (in triplicates) using the semi-automated TMArrayer (Beecher Instruments®, Silver Springs, MD, USA). The thyroid tissue samples investigated, were obtained and maintained in the tissue bank of the A. C. Camargo Hospital.

Immunohistochemistry was performed manually, as follows: Five μm TMA sections were placed on electrically charged slides, de-paraffinised, and rehydrated in decreasing concentrations of alcohol. The endogenous peroxide activity was quenched for 15 min. With H_2_O_2_ and the tissue sections were subjected to heat-induced antigen retrieval in a steamer (90 °C for 30 min), using 10% citrate buffer (10 mM, pH 6.0). Tissues sections were then incubated overnight at 6 °C, with appropriate antibodies. The specification of the antibodies used, the code/clone, the company, the titration, and the antigen retrieval procedure are presented in Table 1. The advanced biotin-free polymer detection system (DAKO, Carpenteria, CA, USA) was used. The chromogen DAB (3.3-diaminobenzidine-tetrahydrochloride; Sigma, St Louis, MA, USA) was applied at room temperature for 5 min. Sections were counterstained with hematoxylin. Positive and negative controls were run in the same batch of reaction, to ensure that the immunohistochemistry reactions are evenly developed to assure their comparability (Additional file 1: Figure. S1).

**Table 1:** Antibodies, code, company, concentration, and antigen retrieval for the different member of the NEK family

Antibody	Code/clone	Company	Concentration	Antigen retrieval
NEK1	–	In House	8μg/mL	1 mM EDTA, pH 8.0
NEK2	SC33167	Santa Cruz	8μg/mL	1 mM EDTA, pH 8.0
NEK3	SC7441	Santa Cruz	8μg/mL	1 mM EDTA, pH 8.0
NEK5	HPA035565	Atlas Antibodies	4μg/mL	1 mM EDTA, pH 8.0

### Immunohistochemistry evaluation

Slides were evaluated by at least two of the authors (TDMH and MBM) and then submitted to other two independent experienced pathologists (JV and FAS), both blinded to tumour features, for the final score. The analysis was performed in two different ways: a) by visual (semiquantitative) and b) by the Aperio’s Image Scope Viewer software (quantitative). For semiquantitative analysis, an individual evaluation of each marker was performed for each spot tissue, by estimating the number of positive cells per TMA spot.

### Semiquantiative evaluation

For semiquantitative evaluation,the multiple tissues were manually scored for each marker and each spot tissue, through the percentage of negative, weak, medium and strong, of total cells, times 0, 1, 2 and 3. The total score for each spot was obtained through the sum of negative + 0, weak + 1, medium + 2 and strong + 3.

For the thyroid cancer patients, the semiquantitative evaluation consisted of estimating the percentage of positive tumour cells and the staining intensity for cytoplasm. For the nuclei, only the percentage of positive cells was measured. The percentage of positive cells was graded like: 0 = negative cell; 1 = up to 25% positive cells; 2 = 25 to 50%; 3 = 50 to 75% and 4 = more than 75% positive cells. Intensity was graded like: 0 = negative; 1 = faint; 2 = moderate and 3 = strong staining.

A final score was calculated adding both percentages of positive cells and intensity of staining, which ranged from 0 to 7.Cases scored from 0 to 2 were grouped as negative (low/decreased expression) and cases scored from 3 to 7 were considered positive (high/increased expression).

The analyses of cytoplasmic and nuclear protein expression were performed only for NEK1 and NEK5, because these are proteins with activity in both of these cellular compartments.

NEK3, on the other hand, is located only in the cytoplasm, as demonstrated in Additional file 2: Figure. S2, and documented in the literature.

### Quantitative evaluation

In addition to semiquantitative evaluation, we further analyzed the immunohistochemical expression using the Aperio Scan Scope slide scanner (Vista, CA, USA). The obtained digital slides were examined using the Aperio’s Image Scope Viewer software, a numerical value proportional to the intensity and extension of brown staining was attributed by the computer analysis, using the formula: Score = (0*% Negative) + (1*% weak positive) + (2*% moderate positive) + (3*% strong positive). The final value of this quantitative analysis was expressed as the mean of triplicates and quadruplicates.

### Statistical analysis

The statistical analysis was carried out using the SAS System for Windows (Statistical Analysis System, version 9.1.3, Service Pack 3 Institute Inc., 2002–2003, Cary, NC, USA). Recurrence-free survival was calculated using Kaplan–Meier survival curves with a log-rank comparison. The nonparametric analysis was performed using either the chi-square or Fisher’s exact test, as indicated. The Mann– Whitney tests were used to compare continuous or arranged measures between two groups; the Kruskal–Wallis test was used to compare three or more groups. The accuracy of NEK1, NEK3 and NEK5 expression to predict malignancy was evaluated using a receiver operating curve (ROC) analysis based on predicted probabilities from logistic regression models. Data analysis was performed with GraphPad Prism (GraphPad Software). All tests were conducted at the significance level *p* ≤ 0.05.

## Results

### NEK expression in human normal and malignant tissues

There are around 520 human protein kinases described and just a small part of them, including the NEK family, have been subjected to studies by the pharmaceutical industry and research institutions [20]. Members of the NEK family seem attractive drug targets because they are associated with target pathway related to cancer development, such as DNA Damage Checkpoint and microtubule functions [21].

To study more broadly the NEK expression profile in cancer, the tissue microarray (TMA) strategy was used in this work. To evaluate the expression profile of NEK1, NEK2, NEK3, and NEK5 in different types of cancer an immunohistochemistry assay was conducted in 10 common types of cancer (esophagus, stomach, colon, lung, thyroid, breast, uterine cervix, pancreas, prostate and kidney) with 4 cases of each and their matched normal tissue. The intensity of brown staining corresponding to the level of NEK expression was measured by semiquantitative (visual; Additional file 3: Table S1) and quantitative method (software ScanScope; Additional file 4: Table S2).

**Fig. 1:**
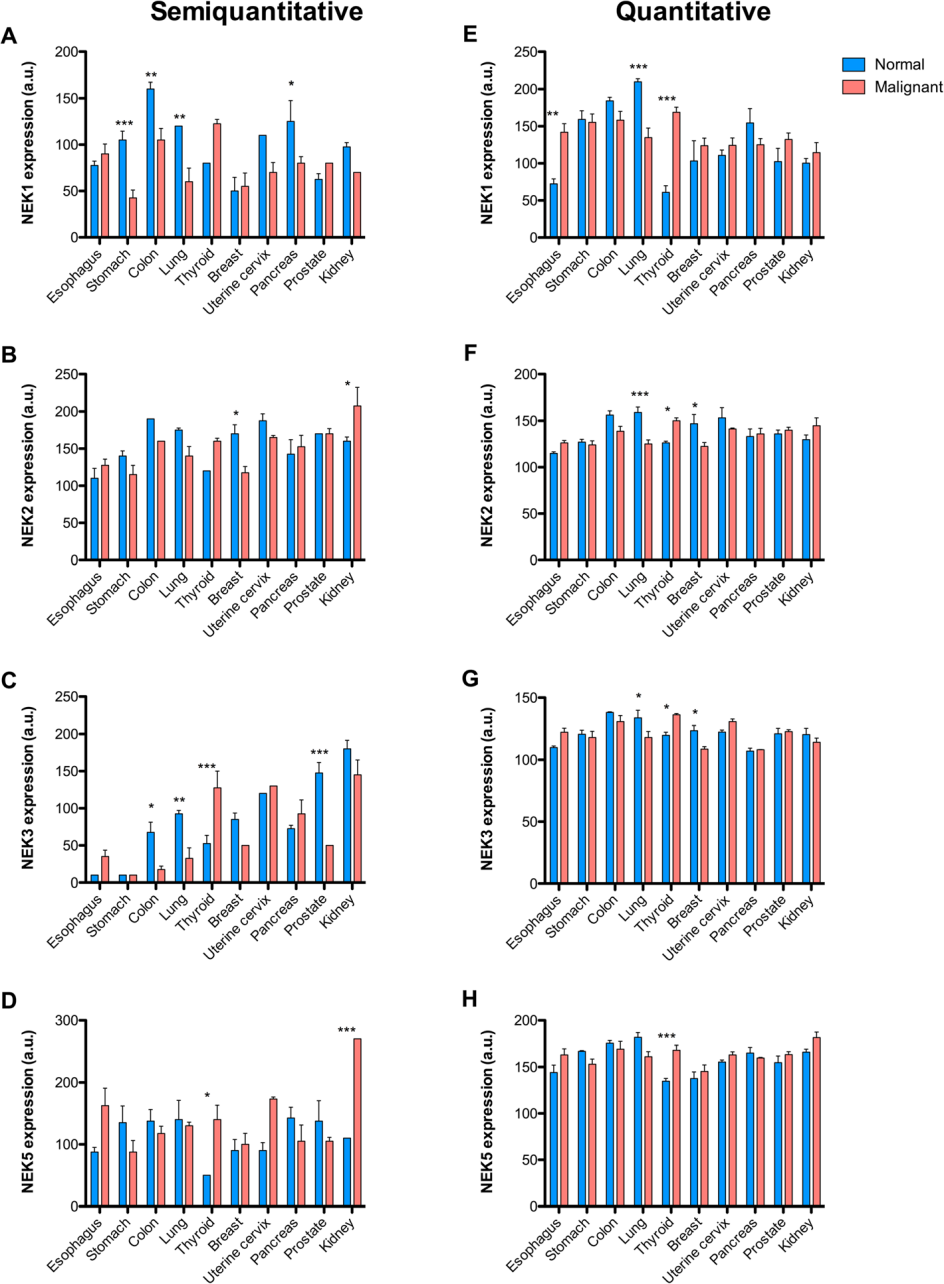
Expression of NEK1, NEK2, NEK3, and NEK5 in normal and malignant tissues. The expression of **a** and **e)** NEK1; **b** and **f)** NEK2; **c** and **g)** NEK3; **d** and **h)** NEK5 was evaluated by immunohistochemistry in normal and malignant tissues of the esophagus, stomach, colon, lung, thyroid, breast, uterine cervix, pancreas, prostate, and kidney. The expression level was calculated through visual (semiquantitative **a**, **b**, **c**, **d**) and ScanScope score (quantitative **e**, **f**, **g**, **h**).Two-Way ANOVA and the Bonferroni post-tests correction were employed for statistical analyses: * = *P* < 0.05; ** = *P* < 0.01; *** = *P* < 0.001

The expression profile of the evaluated NEKs varied considerably between the tissues. For NEK1 we found an increase of expression in normal stomach (*p* < 0.001 for semiquantiative analysis), lung (*p* < 0.01-semiq.; *p* < 0.001-quant.analysis), pancreas (*p* < 0.05 for semiq.), and colon (*p* < 0.01, semiq.), when compared to malignant tissue. On the other hand, in the thyroid, the expression of NEK1 was increased in papillary and medullary carcinoma (*p* < 0.001, quantitative analysis) (Fig. 1 a and e).

Our analysis of NEK2 expression in human tumours showed that in kidney and thyroid the expression of NEK2 is higher in tumour tissues. However, this protein is less expressed in tumour tissues, for the lung (*p* < 0.001 for quantitative) and breast (*p* < 0.05 semiq.; *p* < 0.05 quant.),(Fig. 1 b and f).

NEK3 is increased in normal colon (*p* < 0.05, for semiq.), lung (*p* < 0.01, semiq.; *p* < 0.05 quant.), breast (*p* < 0.05 for quant. analysis), and prostate (*p* < 0.001 for semiq. analysis). However, the thyroid tumour tissue showed significantly higher levels of NEK3 expression when compared to normal tissues (*p* < 0.001-semiq.; *p* < 0.05-quant.), (Fig. 1 c and g).

From the analysis of NEK5, it was possible to observe that the expression profile did not change between normal and tumour tissues, except that the thyroid (p < 0.05 –semiq.; p < 0.001-quant.) and kidney (p < 0.001 for semiq.) had higher levels of expression in the tumour tissues than in the normal cells (Fig. 1 d and h).

**Fig. 2:**
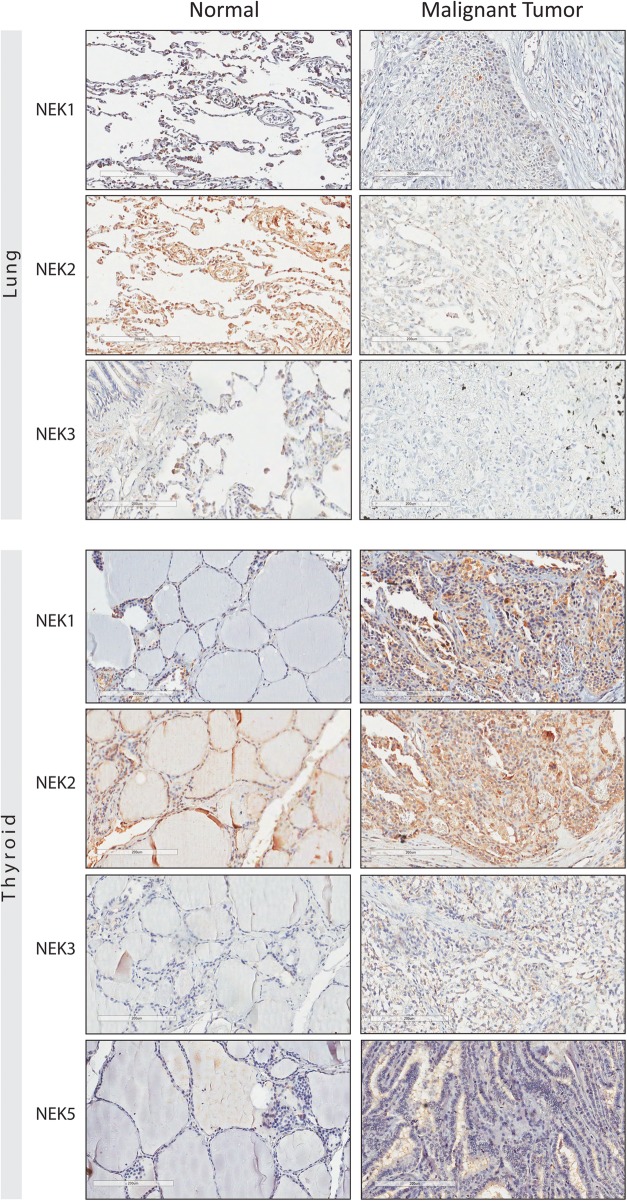
Immunohistochemistry tissue microarray (TMA) of specimens of human normal and malignant lung and thyroid tissue. The immunostaining of the NEK proteins by using the anti-NEK1 (8 μg/mL), NEK2 (8 μg/mL), NEK3 (8 μg/mL) and NEK5 (8 μg/mL) antibodies. The brownish-red region indicates the immunoreactivity of the advanced biotin-free polymer (DAKO, Carpenteria, CA, USA) with the indicated human NEK protein expressed in the tissue of the lung and thyroid, after being labeled with a chromogenic substrate (DAB - Sigma, St Louis, MA, USA)

Both visual and automatic TMA analysis showed that the expression of NEK1, NEK2, and NEK3 was lost during lung malignant transformation. As can be observed in Fig. 2, the difference between normal and malignant lung tissue is more pronounced with the NEK2 expression.

However, in thyroid tissue, the expression of all four: NEK1, NEK2, NEK3 and NEK5 was higher in malignant tissues (Fig. 2). In this case the quantitative analysis showed statistically significant differences for all four NEKs, and the semiquantitative analysis for NEK3 and NEK5.

The TMA results associated with the data from literature about NEKs, suggest, that their function and expression can vary in a tissue-dependent manner. Thus, the expression of the family as a whole needs to be understood in each one of the tissues before thinking about therapeutic strategies.

### Thyroid Cancer patients

In the broad spectrum TMA analysis (semiquantitative and quantitative), we observed an increased expression of NEK1, NEK2, NEK3, and NEK5 in papillary and medullary thyroid carcinoma (Fig. 2 and Additional file 3: Table S1 and Additional file 4: Table S2). However, since this analysis was made with a limited number of patient samples, we expanded the number of cases submitted to TMA analysis. The immunohistochemistry was conducted in samples from 281 patients who underwent thyroid resection (nodules). The expression of NEK1, NEK3, and NEK5 was evaluated by the semiquantitative and quantitative score in the TMA slides. According to semiquantitative analysis, the cytoplasmic expression of NEK1 may differentiate malignant from benign thyroid tissues (*p* < 0.0001) with 94% sensitivity, 47% specificity, 81% positive predictive value (PPV), 74% negative predictive value (NPV). By the quantitative score, NEK1 expression distinguished malignant from benign lesions with 61% sensitivity, 64% specificity, PPV of 79%, NPV of 43%, and accuracy of 62% (mean = 172.97 ± 17.68 vs 164.69 ± 15.86, respectively; *p* = 0.0006) (Table 2).

**Table 2:** NEK1, NEK3, and NEK5 expression levels according to semiquantitative and quantitative IHC analysis in benign and malignant thyroid nodules and nucleus and cytoplasm

Semiquantitative IHC							Quantitative IHC						
Protein expression	Analyzed groups	*p*-value	Sensitivity (%)	Specificity (%)	PPV* (%)	NPV* (%)	Protein expression	Analyzed groups	*p*-value	Sensitivity (%)	Specificity (%)	PPV* (%)	NPV* (%)
Nek1 Cytoplasmic	*Malignant vs Benign*	*<0.0001*	*94*	*47*	*81*	*74*	Nek1	*Malignant vs Benign*	*0.0006*	61	64	79	43
Nek1		*N.S*	*56*	*53*	*75*	*32*							
Nuclear													
Nek3 Cytoplasmic	*Malignant vs Benign*	*N.S*	*98*	*0.7*	*73*	*60*	Nek3	*Malignant vs Benign*	*<0.0001*	78	80	91	60
Nek5 Cytoplasmic	*Malignant vs Benign*	*N.S*	*98*	*0.3*	*75*	*50*	Nek5	*Malignant vs Benign*	*N.S*	77	50	82	42
Nek5		*0.0144*	*26*	*50*	*58*	*20*							
Nuclear													

^a^*PPV* positive predictive value, ^b^*NPV* negative predictive value, and ^c^ *IHC* immunohistochemistry

By the quantitative analysis of NEK3 expression it was possible to differentiate benign from malignant thyroid tissues, with 78% sensitivity, specificity of 80%, PPV of 91%, NPV of 60%, and accuracy of 79% (mean = 188.36 ± 17.11 vs 165.90 ± 14.29, respectively; p < 0.0001). This difference did not appear in the semiquantitative analysis (Table 2).

**Fig. 3:**
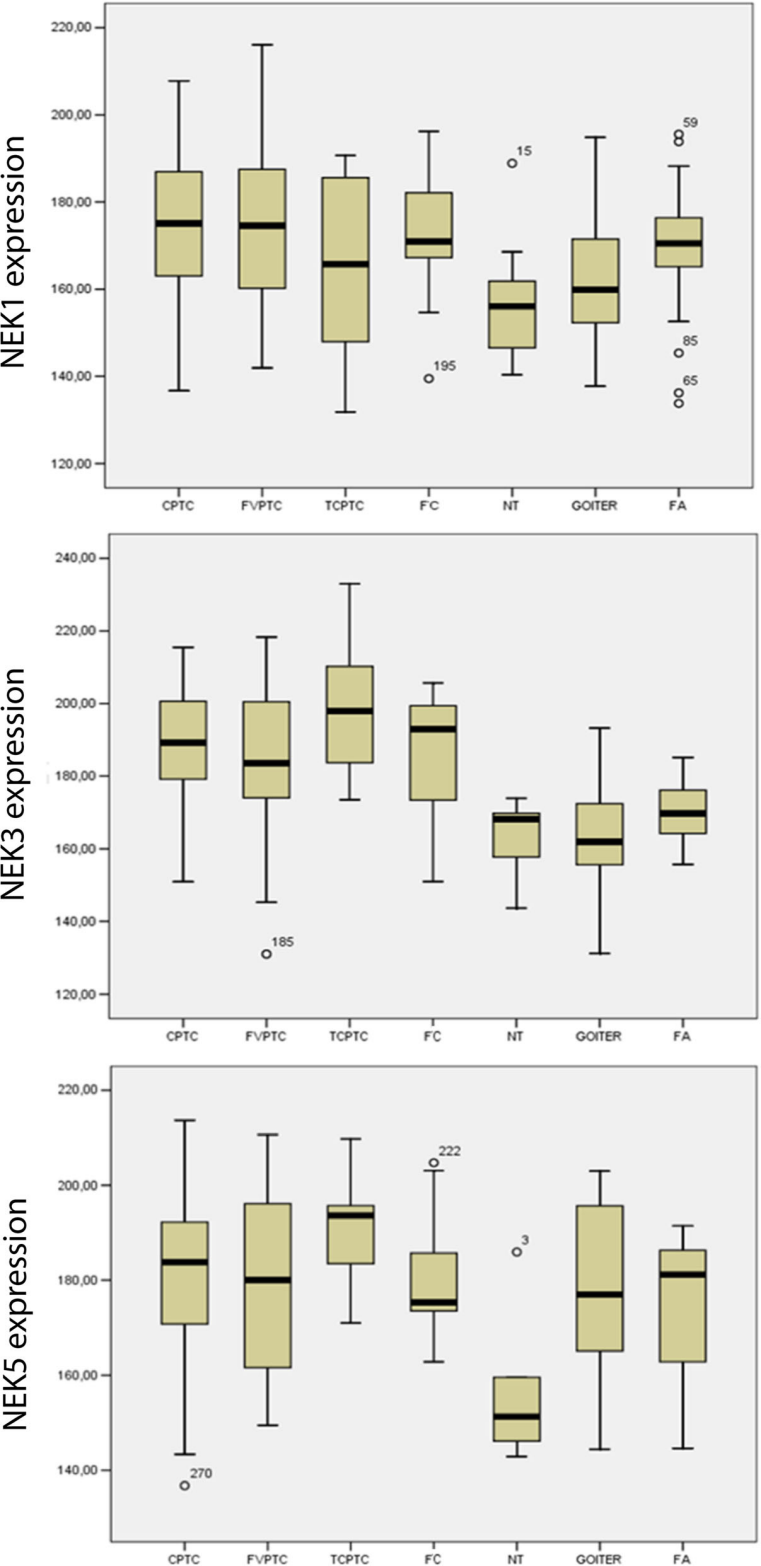
Immunohistochemistry quantitative analyses showing different staining between benign and malignant groups. (A) NEK1 immunohistochemical expression in benign and malignant subtypes of thyroid tissues. (B) NEK3 immunohistochemical expression in benign and malignant subtypes of thyroid tissues. (C) NEK5 immunohistochemical expression in benign and malignant subtypes of thyroid tissues. Data are expressed as mean +/− SEM (standard error of mean). CPTC = classic variant of papillary thyroid carcinoma (107 cases); FVPTC = follicular variant of papillary thyroid carcinomas (48 cases); TCPTC = tall-cell of papillary thyroid carcinoma (13 cases); FC = follicular carcinoma (*n* = 25); NT = normal tissue (*n* = 15); G = goiter (*n* = 42); FA = follicular adenoma (*n* = 31)

Malignant tissue may also be differentiated from benign thyroid tissues by using the NEK5 nuclear expression (*p* = 0.0144), with 26% sensitivity, 50% specificity, 58% PPV, 20% NPV (Table 2). The quantitative analysis showed similar results concerning the differential diagnosis of thyroid lesions for NEKs, wherein the vast majority was higher expressed more in malignant than in benign lesions (Fig. 3 and Table 2).

**Fig. 4:**
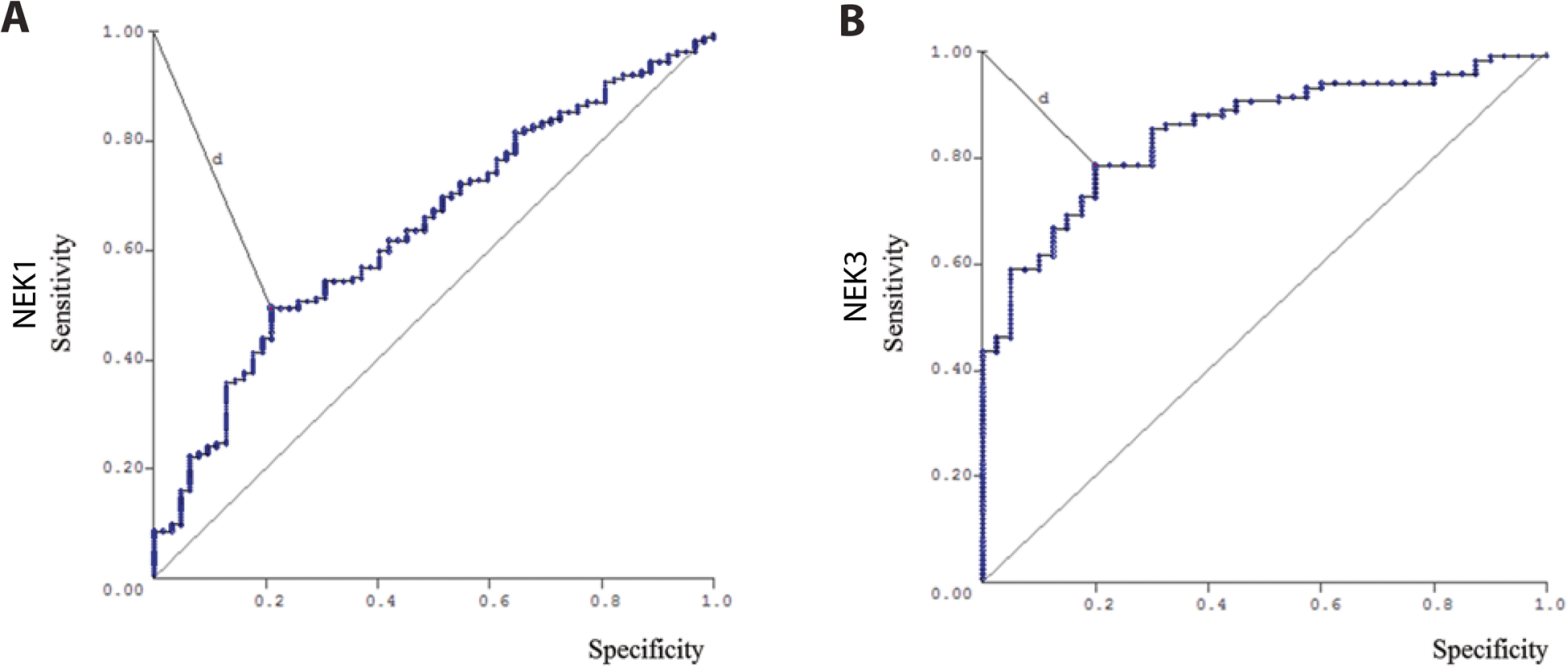
Receiver operating characteristic curve **(ROC).a)** NEK1 and **b)** NEK3 protein expression of patients with DTC versus benign nodules

Aiming to look for NEK1 and NEK3 expressions values to be able to predict malignancy, we further performed a ROC (Receiver Operating Characteristic Curve) analysis based on predicted probabilities from logistic regression models. Using a cut-off of 168.72 for NEK1, we were able to identify malignant nodules with 61% sensitivity, 64% specificity, 79% predictive positive value, 43% predictive negative value, and 62% accuracy (Fig. 4a). The NEK3 ROC curve presented a cut-off point of 176.28 with 78% sensitivity, 80% specificity, 91% predictive positive value, 60% predictive negative value, and 79% accuracy (Fig. 4b).

### The expression of NEKs may be distinct in the thyroid tumour profile

Concerning characteristics as aggressiveness, invasion, tumour size, and multifocality, the quantitative analysis showed that NEK1 expression was higher in tumours in which there was multifocality (175.54 ± 18.82) than in cases without multifocality (170.32 ± 17.45 - *p* = 0.0233); and was more expressed in patients with lymph node metastasis (*p* = 0.0239) (Table 3).

**Table 3:** Relationship between clinical and pathological features of benign and malignant neoplasms of thyroid expressing NEK1, NEK3, and NEK5.* Analysis for Aperio Scan Scope slide scanner

Clinicopathological features	Nek1 Median*	*p*-value	Nek3 Median*	*p*-value	Nek5 Median*	*p*-value
Gender						
Female	172.66	0.4315	188.80	0.2823	181.08	0.3118
Male	171.22		192.35		181.43	
Age						
≤45 years old	172.29	0.3894	188.80	0.2320	183.84	0.1628
>45 years old	173.15		190.63		181.08	
Tumor size						
<2cm	172.29	0.4171	189.20	0.8575	180.88	0.0074
2–4cm	169.50		194.40		174.80	
>4cm	177.83		185.19		195.24	
Extrathyroidal Invasion						
Yes	168.85	0.3437	188.41	0.1023	185.88	0.0424
No	174.33		193.15		180.32	
Capsulation						
Yes	174.98	0.4361	194.87	0.0638	176.06	0.0783
No	174.98		183.39		186.38	
Multifocality						
Yes	175.46	0.0233	189.25	0.3469	182.97	0.4222
No	170.32		193.02		181.29	
Metastasis at diagnosis						
Present	175.43	0.3648	193.29	0.2473	189.71	0.0108
Absent	173.77		188.85		179.76	
Metastasis at evolution						
Present	174.84	0.2053	196.97	<0.0001	178.43	0.0572
Absent	172.21		183.39		183.67	
Lymph node metastasis						
Present	179.22	0.0239	194.40	0.3475	184.50	0.0921
Absent	167.63		197.23		176.05	
TNM						
I	173.81	0.3927	186.34	0.0023	181.56	0.8217
II	168.72		202.86		174.75	
III	180.43		189.86		185.22	
IV	169.83		193.45		182.01	

Considering the quantitative NEK3 expression it was possible to observe that its expression is stronger in patients with stage II (202.86 ± 14.27) when compared with stage I-TMN (186.34 ± 16.71 - *p* = 0.0023) and in patients with metastasis at evolution (*p* < 0.0001) (Table 3).

NEK5 quantitative analysis on the other hand showed high expression in patients with invasion (*p* = 0.0424) and metastasis (*p* = 0.0108); also NEK5 was more expressed in patients with larger tumours (tumour size > 4 cm; 195.24 ± 15.01) when compared with < 2 cm (180.88 ± 17.56 - *p* = 0.0053) and with 2-4 cm (174.80 ± 15.51 - *p* = 0.0014) (Table 3). Parameters such as age, sex and capsule did not present statistical value for the expression of analyzed NEKs.

The Kaplan-Meier analysis demonstrated no correlation between patient’s disease-free interval with NEK1, 3, and 5. Patients with differentiated thyroid cancer were predominantly female (73%) with a mean age at diagnosis of 45.63 ± 15.34 years (range: 15–88 years), as expected, however, no correlation was obtained between NEKs and gender, age or capsulation. These analyses were performed with the quantitative expressions.

## Discussion

Previous studies have demonstrated that NEKs are related to cancer. Many members are related to different aspects of the Hallmarks of Cancer [22]. For example, NEKs 1, 2, 5, 6, 7, 9 and 11 are related to resistance to cell death [23–30] and NEKs 1, 2, 4, 6, 8, 10 and 11 are related to genome instability and mutation [31–37]. Different groups have studied each member of this protein family separately and have observed that the expression of many of them is altered in cancers tissues. The expression of NEK2, NEK6, and NEK11 were related to colorectal cancer [30, 38–40], NEK2, NEK3, NEK5, NEK6, and NEK8 to breast cancer [11, 41–45], and NEK2 and NEK6 to prostate cancer [16, 46].

Genome wide DNA sequencing studies of cancer samples, identified mutations in the coding region of the protein for several members of the NEKs [31, 36, 37, 47, 48], suggesting that such mutations may provide selective advantages for survival and growth of cancer cells. There are few studies with the protein expression of several NEKs and different types of cancer. Based on that, this study focused on the TMA analysis of multiple cancers and normal tissues. Significant increases in the protein expression levels of NEK1, NEK2, NEK3 and NEK5, were found in colon and lung in the normal vs. cancer tissues. These results suggest that the role of NEKs are tissue-dependent.

NEK5 is the least studied member of the family, but the reduced information may not reflect its importance, because NEK5 has been related to many hallmarks of cancer, including the context of cell death [29, 49]. No information is available about the protein levels of NEK5 in cancer and normal cells. Based on that, the expression of NEK5 was found to be higher in normal ciliated tissues such as colon, lung, and kidney. In contrast, in thyroid and kidney carcinoma the level of NEK5 was higher than in normal tissues.

Furthermore, other NEKs showed high levels of expression in thyroid and kidney cancers (Figs. 1 to 3). Loss of function mutations in NEK1 are causal in the case of the murine polycystic kidney disease model lines KAT and KAT2J [34] and one of the key features of the polycystic kidney diseases is aberrant cell proliferation. In contrast the defects in the NEK1 expression can also cause excessive apoptosis [50]. Chromosomal abnormalities in the NEK1 locus were found in Wilms tumour, the most common cancer of the kidney in infants and children [9].

The over-expression of NEK2 was observed in 4 of the 6 highly malignant papillary renal cell carcinomas with chromosome 1q duplication [51]. There may be a relation between the kidneys and the thyroid, since the thyroid hormones can affect renal development and physiology by increasing the renal blood flow and glomerular filtration rate [52]. Due to genetic predisposition and/or treatment, patients with thyroid cancer have an increased predisposition to develop renal cell carcinoma [53]. Similarly, renal carcinoma can metastasize to the thyroid and vice versa [52].

Other authors previously explored the role of NEKs in the kidney, but until now, there are no reports in the literature of the involvement of the NEKs with TC. Once NEK1, NEK2, NEK3, and NEK5, all showed a higher level of expression in thyroid tumour tissue it was decided to expand the number of thyroid cases submitted to TMA analysis. TC is the most common endocrine malignancy and its incidence and mortality rates differ based on the histological subtypes [54]. During the progression of Differentiated Thyroid Cancer (DTC), the most frequent and relevant molecular alterations comprise rearrangements of tyrosine kinase receptor genes, such as RET/ PTC and NTRK1 (neurotrophic receptor tyrosine kinase 1), or protein-activating point mutations affect the cellular responses to signals of growth and differentiation signaling pathways, including: RAS, BRAF, PI3K, and oncogenic fusion protein PAX8-PPAR [55, 56].

In this work, it was demonstrated that NEK1 and NEK3 have different expression levels when comparing malignant and benign thyroid tissue. NEK1 was overexpressed in CPTC and FVPTC and NEK3 in TCPTC followed the FC. We also observed that NEKs 1, 3 and 5 expression levels were related to aggressiveness characteristics such as multifocality, invasion, metastasis, TNM and tumour size of patients with thyroid cancer. Previously, Zhu and colleagues [57] demonstrated that NEK1 is upregulated in glioma and correlated with the proliferation marker (Ki-67), tumour grade and patients’ poor survival [57]. In a screen for potential anticancer drugs in PTC, the NEKs 1, 2 and 11 were pointed as candidates target [19].

In the present work, we demonstrate that the expression of NEK1 might be used as a predictive factor for lymph node metastasis in patients with DTC (Differentiated thyroid cancer). In a PTC (Papillary thyroid carcinoma) lymph node metastasis mouse model, tissue microarray data showed that simultaneous over-expression of protein kinase Aurora-A and CFL-1 (a regulator of actin polymerization) correlated with lymph node metastasis in thyroid cancer tissue [58].

The follicular pattern is one of the major obstacles to differentiate the diagnosis of thyroid lesions, including follicular adenoma (AF), follicular carcinoma (CF) and follicular variant of papillary thyroid carcinoma (FVPTC) [59]. Especially in the discrimination of minimally invasive follicular carcinoma of follicular adenoma and correct diagnosis of follicular carcinoma [60, 61]. Efforts are directed to search for novel immunohistochemical markers for the diagnosis and prognosis of thyroid lesions, especially those of follicular patterns. Promissing candidates are: CD56, galectin-3 (Gal-3), CK19 and mesectorial Hector Bat-tifora − 1 (HBME-1), but so far none of these have been entirely satisfactory [62, 63].

Lung carcinomas are classified into non-small cell carcinomas (CNPCs) and small cell carcinomas (NSCLC) [64] with CNPCs being the most common (75–80%) [65]. However, because of markedly different prognostic and treatment implications among different lung tumours, a better molecular characterization is required, especially for the most common NSCLC types and lung adenocarcinoma.

In summary, these data suggest that NEK expression levels can be explored as important biomarkers of cancer prognoses and probably as new molecular targets in cancer therapy. In this work, we demonstrated that NEK expression and functions are tissue-dependent, and that their expression level are altered when comparing normal and cancer cells.

## Conclusions

The TMA results, which encompassed different histological types of cancer studied, suggest that the expression and function of NEKs are tissue-dependent. Regarding mainly the expression of NEK1 and NEK3 in DTC, our results indicate that these NEKs may have an important role in thyroid malignancies, allowing to identify malignancy and aggressiveness features of DTC cases.

## Supplementary information

**Additional file 1: Figure. S1.** Controls of immunohistochemistry reaction. Malignant melanoma was used as tissue marker, (positive staining) for A) NEK1, B) NEK2, C) NEK3 and D) NEK5. E) Normal skin in the absence of primary antibody was used as the negative control. The scale bar = 100 μm.

**Additional file 2: Figure. S2.** NEKs expression in different lesions thyroid: (a1) NEK1 negative (× 400); (a2) NEK1 positive (× 400); (b1) NEK3 negative (× 400); (b2) NEK3 positive; (c1) NEK5 negative (× 400); (c2) NEK5 positive and d(1) NEK6 negative (× 400); (d2) NEK6 positive (× 400).

**Additional file 3: Table S1.** Semiquantitative expression patterns of NEK1, NEK2, NEK3, and NEK5 in tissue microarrays comparing benign and malignant neoplasms tissue of Esophagus, Stomach, Colon, Lung, Thyroid, Breast, Uterine cervix, Pancreas, Prostate, and Kidney.

**Additional file 4: Table S2.** Quantitative expression patterns of NEK1, NEK2, NEK3, and NEK5 in tissue microarrays comparing benign and malignant neoplasms tissue of Esophagus, Stomach, Colon, Lung, Thyroid, Breast, Uterine cervix, Pancreas, Prostate, and Kidney.

## Data Availability

The data here presented are summarized in Methods section. The complete dataset can be retrieved from the authors upon formal request of interested readers.

## Abbreviations

AC: Snaplastic thyroid cancer
CPTC: Classic variant of papillary thyroid carcinomas
DTC: Differentiated Thyroid Cancer
FA: Follicular adenomas
FC: Follicular carcinoma of the thyroid
FTC: Follicular thyroid carcinomas
FVPTC: Follicular variant of papillary thyroid carcinomas
G: Goiters
IHC: Immunohistochemistry
MC: Medullary thyroid carcinomas
NEKs: NIMA-related kinases
NPV: Negative predictive value
PC: Well-differentiated papillary thyroid tumours
PK: Protein kinases
PPV: Positive predictive value
PTC: Papillary thyroid carcinomas
ROC: Receiver operating curve
TC: Thyroid cancer
TCPTC: Tall-cell of papillary thyroid carcinomas
TMA: Tissues microarray
TNM: System of Classification of Malignant Tumours
