# Supplementary figures and images for: Expression of the NEK family in normal and cancer tissue: an immunohistochemical study
Source: BMC Cancer. 2020 Jan 6;20:23. doi: 10.1186/s12885-019-6408-4 (PMC6945616; doi:10.1186/s12885-019-6408-4)

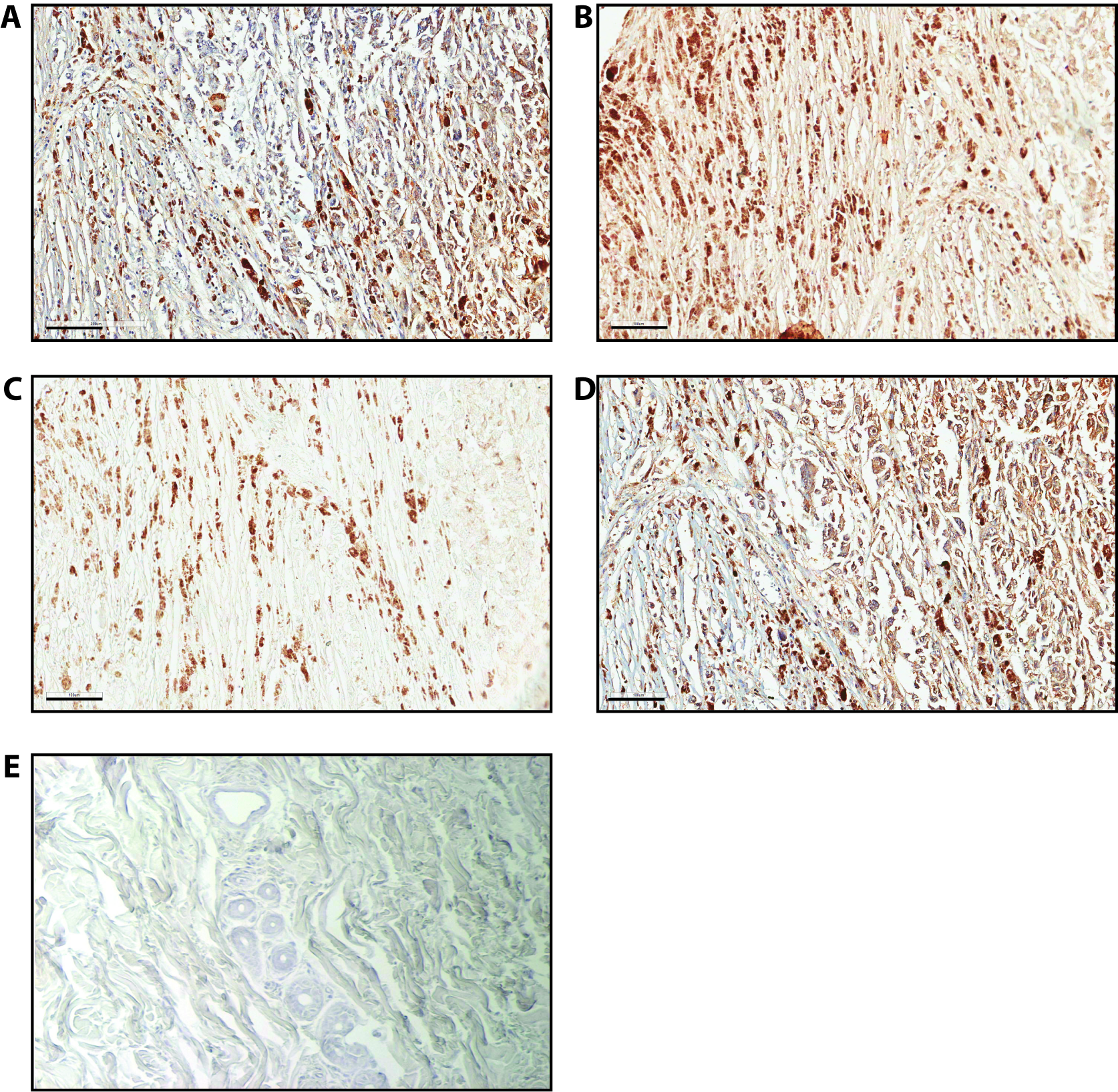

Supplement: Supplementary file 1 — Additional file 1: Figure. S1. Controls of immunohistochemistry reaction. Malignant melanoma was used as tissue marker, (positive staining) for A) NEK1, B) NEK2, C) NEK3 and D) NEK5. E) Normal skin in the absence of primary antibody was used as the negative control. The scale bar = 100 μm. [file 12885_2019_6408_MOESM1_ESM.tif]

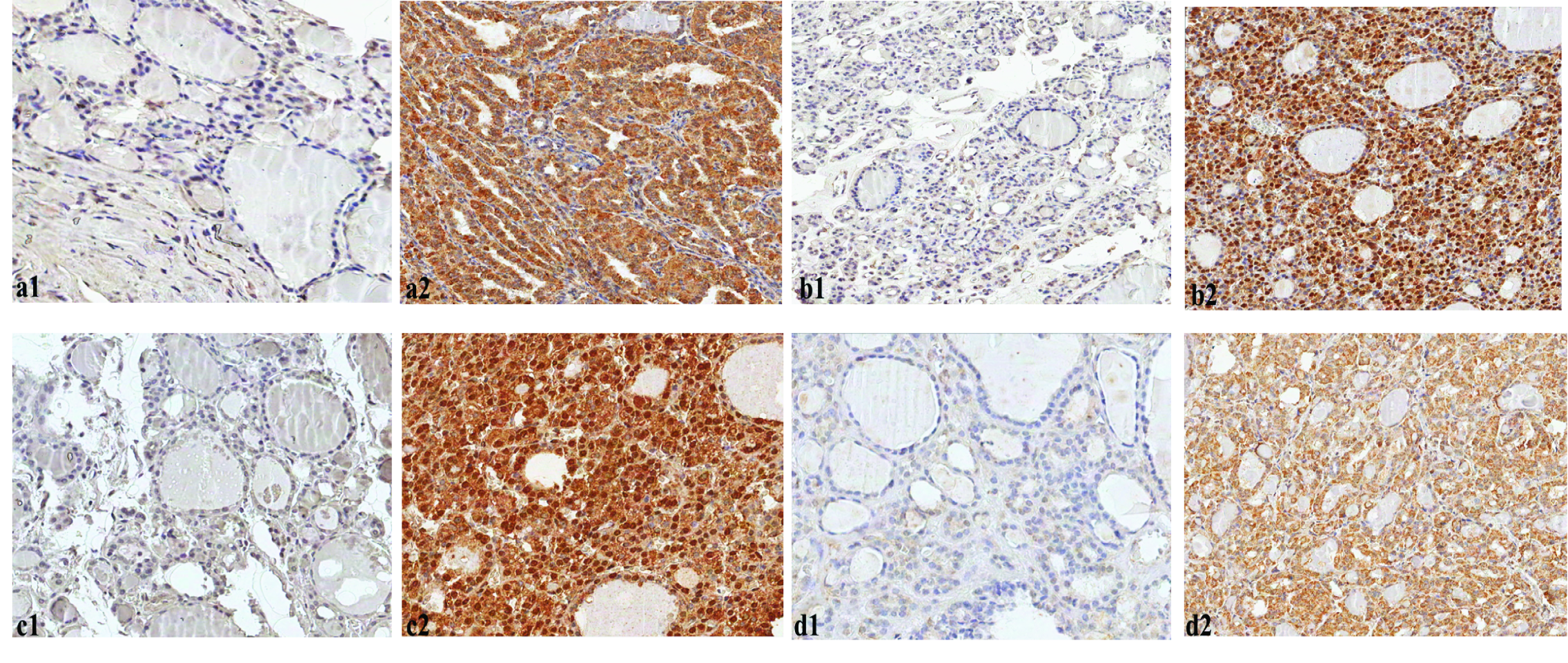

Supplement: Supplementary file 2 — Additional file 2: Figure. S2. NEKs expression in different lesions thyroid: (a1) NEK1 negative (× 400); (a2) NEK1 positive (× 400); (b1) NEK3 negative (× 400); (b2) NEK3 positive; (c1) NEK5 negative (× 400); (c2) NEK5 positive and d(1) NEK6 negative (× 400); (d2) NEK6 positive (× 400). [file 12885_2019_6408_MOESM2_ESM.tif]
